# Autumnal Diseases

**Published:** 1867-09

**Authors:** 


### HALL'S JOURNAL OF HEALTH.

Our Legitimate Scope is almost boundless; for whatever begets pleasurable and harmless feelings, promotes Health; and whatever induces disagreeable sensations, engenders Disease.

WE AIM TO SHOW HOW DISEASE MAY BE AVOIDED, AND THAT IT IS BEST, WHEN SICKNESS COMES, TO TAKE NO MEDICINE WITHOUT CONSULTING A PHYSICIAN.

VOL. XIV.] SEPTEMBER, 1867. [No. 9.

### AUTUMNAL DISEASES.

THESE are chiefly diarrhœa, dysentery and various grades of fevers, from slight 'creeps' to congestive chills, for fever is the reaction of coldness, but when there is not power enough in the system to react from the cold stage, death is certain, as in congestive chill, in which the blood becomes so cold, so thick and so impure that it ceases to circulate, becomes stagnate and the machinery of life stops forever. Hundreds of thousands die every autumn of the three forms of disease mentioned, but not one need die, they are avoidable diseases, their causes being known and all that is required is to bring a very moderate amount of intelligence to bear iu avoiding those causes. A baby will avoid putting its finger in the candle a second time; it remains only to grown-up stupids to expose themselves to the causes of disease year after year and thus recklessly imperil health and even life itself.

The cause of Autumnal diseases is an emanation from the surface of the earth in those localities where are found in combination heat, moisture and vegetable matter such as leaves, wood, &c., for the heat of eighty degrees combined with moisture induces decay, and from this decaying substance something arises which if breathed or otherwise taken into the system induces the diseases mentioned, sooner or later.

What this emanation is, has hitherto been merely a conjecture because it was so impalpable, so like thin air, that the atmosphere which contained it when subjected to chemical analysis yielded nothing beyond the constituents of pure air. But within a year or two it has been ascertained that if a quantity of air of a miasmatic locality is bottled up and is conveyed to a sleeping apartment, the person who breathes it will, in a short time, have more or less decided symptoms of fever and ague; and on examining his saliva or the inside of his mouth a living, moving thing is clearly visible with microscopic aid. Observation and experiment have shown incontrovertibly that there are two ways of escaping the ill effects of having these living things introduced in the system,—persons must avoid living in localities where the land is rich, flat and moist, or they must drain those lands; but it is possible to live in such places and have reasonbly good health simply by keeping in the house of mornings, with a brisk blazing fire until breakfast is eaten, and take supper at sundown, because it has been found that these emanations are more poisonous at sunrise and sunset and that if the stomach is excited to action by the process of digestion the emanation is rendered innocuous, perhaps from the fact, in part, that the juices of the stomach at the time of digestion are of a character to destroy the life of these living things; but the fact remains the same, whether this supposition is true or not.

A practical use may be made of this subject in the light of these facts, in reference to breathing night air. Very many advocate the raising of windows in a sleeping apartment summer and winter, all the year round; the theory seems a good one, but experience will not corroborate it. Persons living on water courses where the "bottom lands," as they are called, are rich, luxuriant and damp will save health and life itself by keeping all outside doors and windows opening into chambers closed from sundown to sunrise during the three Autumnal months, in fever and ague or intermittent localities.

WASHING.

Any man will feel more like a man, in the consciousness of having on a clean shirt; and as the hired help who go through the motions of washing our clothing without accomplishing the object, one-half are particularly disposed to shirk labor in these days of hot suns and suffocating atmospheres, it might be well to adopt some plan by which the same amount of labor expended in half washing our clothing could be made to do the thing thoroughly. Such a plan would be of incalculable value to that army of hard working wives whose husbands are tillers of the soil.

That excellent paper, the OHIO CULTIVATOR, comes to the aid of farmers' wives right gallantly and assures them that the washing may almost do itself while the poor tired woman is lying on the lounge looking at it, thus: Take one pound of sal-soda (saleratus or bi-carbonate of soda, all of which are different names for the same article) and a half pound of unslacked lime, put them in a gallon of water and boil twenty minutes, let it stand till cool, then drain off, put in a small jug or jar; soak your dirty clothes over night, or until they are wet through, then wring them out and rub on plenty of soap, and in one boiler of clothes well covered with water, add one teacupful of washing fluid; boil half an hour briskly, then wash them thoroughly through one suds, rinse, and your old clothes will look better than the old way of washing twice before boiling.

FEEDING THE SICK.

An intelligent officer in the army remarked one day that he had long observed that a larger number of men who were sent home to their friends sick, died than of those who remained in the army. On being asked his opinion as to what was the reason of such a result he said, "Because their friends feed them up so." The answer was undoubtedly the true one. As in health we eat heartily, it seems to be concluded that if a sick person can be made to eat heartily he will get well right away, and to induce them thus to eat, all the delicacies and tempting things that can be thought of are crowded in on the poor laboring stomach, internal fever is excited, no nourishment is drawn out of it, and the invalid consumes away by internal fires

We constantly read of persons horribly wounded in war or otherwise injured, who, unable to obtain human aid, are exposed to rain and night air and other inclemencies and would have starved, but for the roots and berries they crawled about to procure, and yet survived. It is a rule, with very few exceptions, that the sick should not have their appetites tempted; that they should wait until they felt as if plain bread and butter would taste good to them, and even then, not eat at less than five hours interval.

Tho stomach is weak like every other part of the body, and to put upon it a task which its instincts do not seek, is unwise and illogical in the highest degree. You can't make a sick pig eat, while man, a bigger pig, "forces" the food upon himself when he has not the slightest inclination and even takes measures to create the inclination. Nor bird, nor beast, nor creeping thing will eat when sick, but man, the biggest brute of all, will.

One plain wholesome dish for an invalid is prepared thus—Take some common Indian corn, roast it as you would coffee, grind it in a coffee mill and make it, in the usual way, into a mush or gruel, or make it into thin cakes nicely browned and eat either cold or hot, alone or with sugar or salt or syrup or butter, in whatever way the stomach will receive it most kindly and retain it. This parched corn meal or parched rice, boiled in sweet milk, is one of the best non medicinal remedies known for the relief of diarrhœa or even dysentery, if the patient will remain quietly in bed for a day or two. This is especially valuable for children suffering with bowel complaint.

Another excellent article of food, wholesome and nutritious, is common wheat cleaned and washed, then let it be soaked in warm water and when the grains have softened and swollen, boil slowly until soft enough to be eaten with milk or sugar or syrup, according to the taste; it may be salted a little; if any is left over it may be cut in thin slices and then fried like mush.

It is well known among physiologists that the teeth and bones are durable and strong in proportion as they contain one of the chemical constituents of lime, and that the food which contains these constituents in large quantities is best adapted to the formation of good teeth and strong limbs. In the item of bread, used in every family, a striking fact is exhibited: in 500 pounds of the finest flour for table use there are thirty pounds of these bone-forming elements; in an equal amount of bread made of wheat, or prepared as above, there are eighty-five pounds of the bone and tooth-forming principles, hence it is not to be wondered at, that the Scotch are the thriftiest and hardiest race in the world, for they luxuriate on their dearly beloved oat-meal gruel, bread and cakes three times a day. The whole grain of Indian corn or wheat prepared as recommended does not fatten as much as fine flour, the latter having twice the amount of fat-forming principle; but fat is not strength; it does not give endurance, toughness, hardness, capability of work; the whole grain of the Indian, wheat, rye, oats, does, and from five to fifteen, children should be compelled to make one daily meal, wholly, of one of these grains, prepared as above.

### COLD IN THE HEAD.

When a person takes a cold it will "settle" in the head, throat, chest, bowels, or joints, according to circumstances; if in the head, inducing an unpleasant "stuffiing up" and an interruption of the sense of smell. An immediate and grateful relief is experienced sometimes by applying a smelling-bottle (hartshorn) to the nose and keeping it there until it begins to be felt, then remove the bottle for a moment and reapply as before; this is repeated seven or eight times in the course of a few minutes—the nostrils are freed and the sense of smell restored. This same hartshorn gives almost instant relief from the effects of the poisonous bites of all insects, vermin and reptiles by bathing the parts bitten, very freely.

